# Unravelling a diversity of cellular structures and aggregation dynamics during the early development of *Myxococcus xanthus*

**DOI:** 10.1098/rsbl.2024.0360

**Published:** 2024-10-23

**Authors:** Natsuko Rivera-Yoshida, Alejandro V. Arzola, Mariana Benítez

**Affiliations:** ^1^ Laboratorio Nacional de Ciencias de la Sostenibilidad, Instituto de Ecología, Universidad Nacional Autónoma de México, Ciudad de México C.P. 04350, Mexico; ^2^ Departamento de Física Cuántica y Fotónica, Instituto de Física, Universidad Nacional Autónoma de México, Ciudad de México C.P. 04350, Mexico

**Keywords:** multicellular development, cellular structures, cellular density, aggregation, *Myxococcus xanthus*

## Abstract

Aggregation underlies the collective dynamics of a diversity of organisms, enabling the formation of complex structures and emergent behaviours on interaction with the environment. Cellular aggregation constitutes one of the routes to collective motility and multicellular development. *Myxococcus xanthus*, a social bacterium, is a valuable model for studying the aggregative path to multicellularity, a major transition in the evolutionary history of life. While the collective developmental behaviour of *M. xanthus* has been largely studied in high cellular densities, there is a lack of understanding at low-density conditions that can be ecologically relevant. In this work, we study the early stages of emergent collective behaviour of *M. xanthus* under nutrient-poor and low-density conditions, uncovering the formation of diverse cellular structures with different shapes and sizes, ranging from individual cells to networks comprising thousands of cells. We study their motility patterns and their prevalence along development and discuss their cross-scale role on the population’s exploratory dynamics. This work contributes to understanding key, yet largely understudied, aspects in the early stages of multicellular development in myxobacteria, shedding light on the dynamics underlying aggregative processes in this and other taxa and study systems.

## 1. Introduction

Aggregation is a ubiquitous phenomenon of non-equilibrium systems that leads to the emergence of spatial structures both in living and non-living matter [1–5]. In particular, aggregation among self-propelled particles in the microscopic world arises from their diverse attractive interactions and confinement, exhibiting structures that vary in size and shape across spatiotemporal scales [6]. Cellular aggregation constitutes one of the routes to collective motility and multicellular development, and understanding it can shed light on the ecological and evolutionary processes underlying the formation of biofilms and multicellular structures in diverse prokaryote and eukaryote lineages [7,8]. *Myxococcus xanthus*, a motile, rod-shaped bacterium that glides across surfaces, exhibits different collective behaviours under nutrient-availability conditions. Under starvation, *M. xanthus* cells undergo aggregation through a developmental process that can culminate in the formation of differentiated multicellular structures known as fruiting bodies [9–11]. Because of this, *M. xanthus* serves as a valuable model system to study the aggregative path to multicellularity, a major transition in the evolutionary history of life [12,13].

*Myxococcus xanthus* aggregation exhibits a remarkable richness of cellular structures and behaviours depending in part on the cellular density [14–16]. These have been well-studied at high, standard laboratory densities, at which cells form dense mats that completely cover the substrate, tend to remain in contact and align in a regime that mimics a nematic liquid [15]. Aggregation dynamics are also driven by cellular reversal timing and correlated with the nearest aggregate size, which, in turn, correlates with local cell density [15,17]. In a *M. xanthus* mutant that is unable to reverse and has been studied at nutrient-rich conditions, collective motion emerges only above a density value, allowing the formation of large structures [14]. However, there is no systematic account of how *M. xanthus* cells at low densities and in the developmental regime (nutrient-poor conditions) organize and transit from single cells to cellular structures, especially during the early stages of development when cells are beginning to interact with themselves and the substrate. Addressing this gap may uncover aspects of aggregation that are impossible to detect at higher cellular densities but might be critical at the onset of development.

In this study, we describe and analyse the organization of populations of *M. xanthus* cells in nutrient-poor and low-packing density conditions during the initial 8 h of development. Our experimental set-up allowed us to acquire image and video for large areas of up to 1000 × 624 μm. We explore how cellular organization changes with cellular density and developmental time. With this, we contribute to unpacking and understanding largely understudied stages and processes in the development of multicellular structures in myxobacteria and to further understanding aggregation as an ubiquitous phenomenon in nature.

## 2. Methods

### (a) Experimental growth and developmental conditions

Following Yang & Higgs [18], DK1622 strain was taken from a frozen stock by spotting 50 μl onto a casitone yeast extract (CYE) agar plate (1% Bacto Casitone, 10 mM tris–HCl (pH 7.6), 0.5% yeast extract, 10 mM MOPS (pH 7.6) and 4 mM MgSO_4_; BD Bacto™ Agar) and incubated at 32°C for 2 days. Cells from the resulting colony were transferred to 25 ml of CYE liquid medium and incubated at 32°C, shaking at 250 rpm overnight. The culture was diluted to 0.7 OD550 (nutrient-rich liquid culture).

For the development assays, cells were harvested by spinning them at 8000 rpm for 5 min. The pellet was washed with a tris phosphate magnesium (TPM) medium solution (10 mM tris–HCl (pH 7.6), 1 mM K_2_HPO_4_ and 8 mM MgSO_4_) and resuspended in 1/10 of the original volume. This cellular stock was diluted 1 : 350, 1 : 35 and 1 : 10. Fifteen microlitres of each dilution were spotted onto 5 ml TPM agar plates (BD Bacto Agar, 2.4%) and incubated at 28°C for 8 h. The essays were repeated three times for statistical support. When spotted and dried onto agar plates, the cells in the three dilutions exhibit 0.02, 0.1 and 0.23 packing densities, respectively, showing a qualitatively different organization: mostly isolated single cells, crowded single cells and some pairs of cells and sparse small groups of cells, respectively. We will refer to these conditions as low, medium and high density, although they are all relatively low in comparison with the densities usually used in *M. xanthus* essays.

### (b) Microscopic local observation, image and video acquisition and analysis

The samples were observed through TPM agar medium using an inverted optical microscope with a 10× objective lens (Olympus UPlanSApo series, 0.4 NA), illuminated with amber light and recorded with a Basler acA 1600-20uc with an overall resolution of 0.52 μm pixel^−1^ and 1000 × 624 μm field of vision. The microscope was set inside an isolated chamber with a controlled temperature of 28°C. Micrographs were taken for two of the three replicates, while 15 min videos were taken at *t* = 0, 4 and 8 h from the onset of starvation (after the spots dried) for the third replica at an acquisition rate of 1 frame s^−1^. Micrographs and video frames were binarized using segmentation through edge-detection and intensity-threshold criteria implemented with the Image Processing Toolbox of MATLAB. Analysis from the resulting database was conducted using R v. 4.2.0.

To study the exploration of space at the population level, we defined the fraction of explored area as $a_{t,T}=\Sigma_{i,j}$, with $U_{t,T}=I_{t,0}\cup I_{t,\Delta t}...\cup I_{t,T}$ the union of the binarized images, I, from the developmental time *t* to *t* + *T* taken every Δ*t* = 50 s. Figure 3*e* shows $a_{t,T}$ as a function of the three developmental times (*t* = 0, 4 and 8 h) and *T* = 600 s for cultures with medium and high cell density. (Cells at low density and *t* = 0 s practically do not explore new areas.) The resulting plots reflect the overall changes due to the change in the position, shape and size of the different cellular structures over time.

## 3. Results

### (a) Diverse cellular structures dynamically arise and can coexist along development

**Figure 1.**
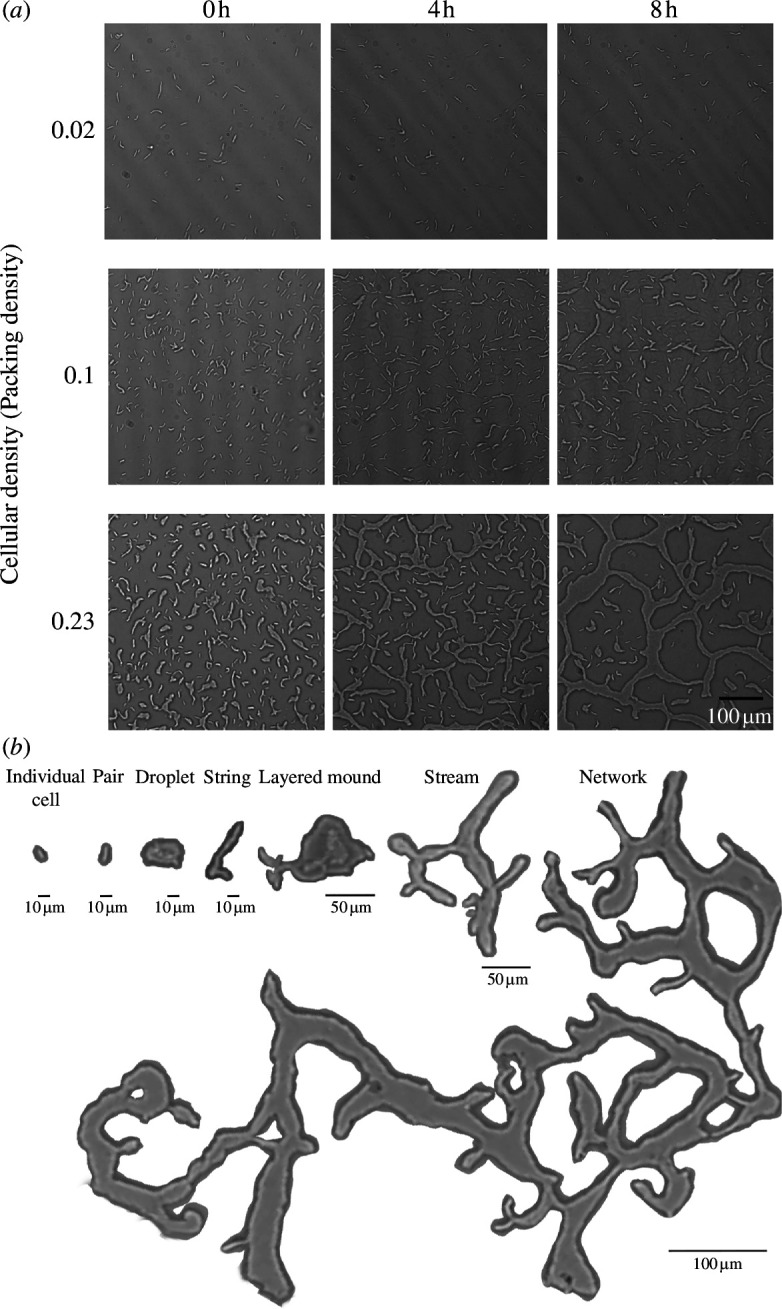
*Myxococcus xanthus* early development (from 0 to 8 h) with varying cellular densities (packing densities). (*a*) At low cellular density, *M. xanthus* cells stay as single cells, pairs or triads through time. At medium cellular density, single cells and small elongated cellular structures coexist in dynamic arrangements in a homogeneous spatial distribution. At high cellular density, single cells and larger cellular structures varying in size and shape coexist in dynamic arrangements. (*b*) Typology of structures with different sizes. Morphologies and behaviours emerge through the continuous merging and dissociation of cells and groups of cells, coexisting along the development.

To explore the effect of cellular density on the early developmental dynamics of *M. xanthus*, we followed the dynamics of cellular structures in the wild-type (WT) DK1622 strain at three different cellular densities (all of them relatively low but referred here as low, medium and high (§2)). Micrographs and 15 min videos were taken at 0, 4 and 8 h for each condition. Through this time-lapse, we monitored the formation and eventual coexistence of motile structures exhibiting different morphologies and a wide range of sizes, and dynamically forming through the continuous merging and dissociation of individual cells and other cellular structures (figure 1*a*). We first describe these structures qualitatively in terms of their size in number of cells (ranging from one to thousands and from a scale of tens to hundreds of microns), their spatial exploration mode (directed or not), presence of reversals and the stability of their boundaries (see a more detailed and quantitative description in figure 3).

Considering these features, we propose a typology of cellular structures that applies to *M. xanthus* early development at low densities but is not restricted to these conditions (figure 1*b*; electronic supplementary material, videos S1–S9). *Individual cells* tend to move locally, without an apparent target or large net displacement, oscillating back and forth along the trails they form. When arranged in *pairs* through head-to-coil contacts, their motility is qualitatively similar to that of individual cells, but they can come together and split as the cells in the pair reverse. *Strings* are elongated, thin and flexible structures characterized by unstable boundaries and conformed by three to tens of cells. These structures can maintain their elongated form, split, become part of stream or transit into more rounded structures. *Droplets* include a diversity of irregular but overall rounded morphological structures that can include up to hundreds of cells and exhibit rapid and persistent displacement with an apparent target, often merging and dissolving rapidly upon contact with individual cells and other cellular structures. *Layer mounds* are rounded structures composed of two or more layers that can move as a single structure, though the layers can also move independently. Droplets may coalesce into layered mounds or wide-branched *streams*, each comprising hundreds of cells and also exhibiting unsteady boundaries but different in their morphology and behaviour from droplets and strings (figure 1*b*). Within streams, cells appear to align and tend to move collectively, with anisotropic effective motion (electronic supplementary material, video S10), as reported in previous work [15]. *Networks* are branched and large structures that can encompass almost all cells within a sample. While these networks exhibit continuous shape changes and single cells within move (typically aligned), they maintain relatively stable boundaries over extended periods. Overall, cellular structures seem to move more rapidly than individual cells, exploring larger areas of the substrate. While some of these structures and phenomena have been previously described from an active matter perspective, e.g. cell flocks and streams in Thutupalli *et al*. [15], or rafts and peninsulas in the study of cellular motility in the edge of a swarm by Kaiser & Crosby [19], our proposed typology highlights a variety of previously unreported cellular structures and behaviours involved in the early aggregation dynamics of *M. xanthus*.

### (b) The prevalence of cellular structures changes along development and with varying initial densities

**Figure 2.**
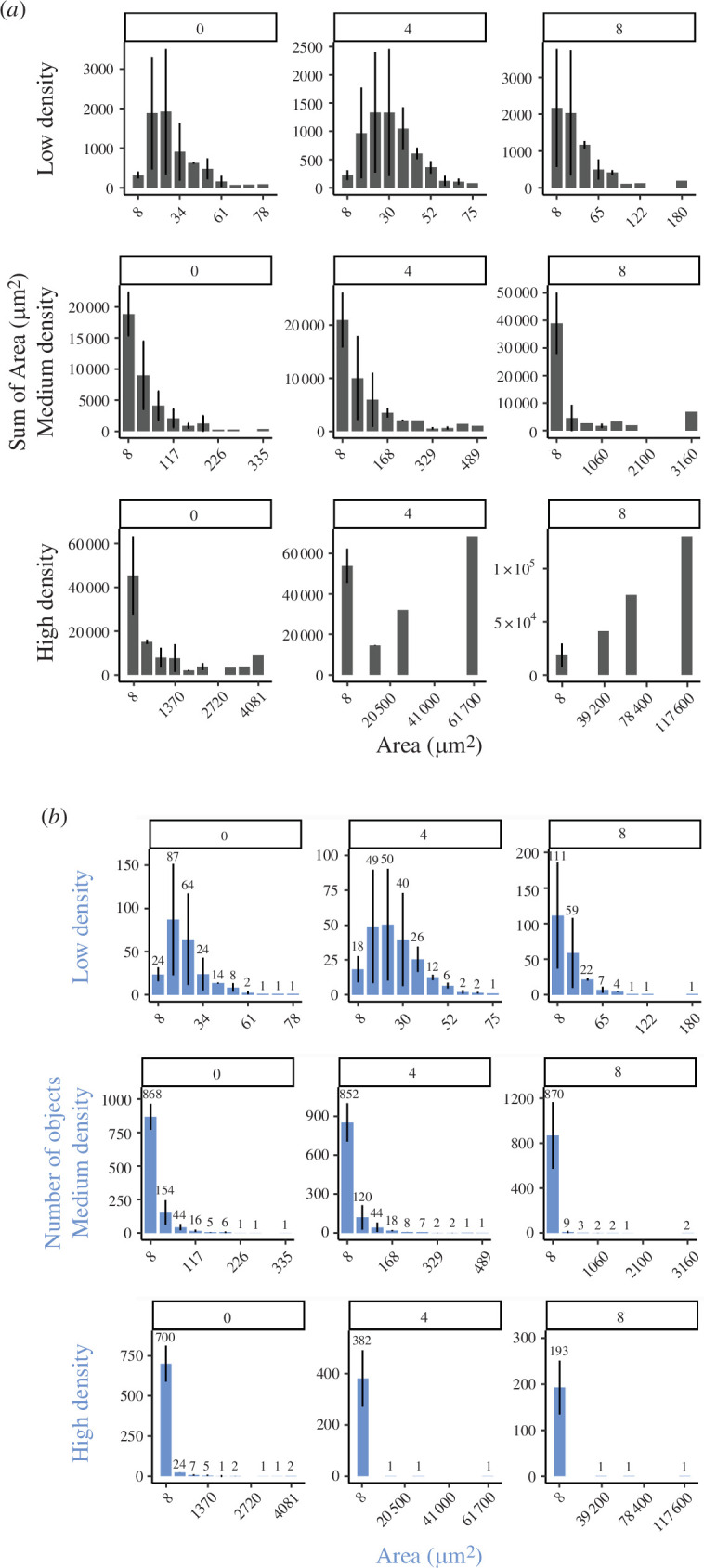
Occupied area and frequency of structure sizes for different initial density conditions throughout early development. (*a*) Occupied area and (*b*) number of objects for different structure sizes. Each bar plot has 10 bins with uniform width in the whole range of values of area. For clarity, we only show the labels of the minimum value for four bins. Data were obtained from three replicates.

We observe that the prevalence of different cellular structures and the overall cellular organization change over time and with initial cellular density (figure 2). To better understand how these changes occur, we quantified the size and number of the cellular structures present at 0, 4 and 8 h.

At low cellular density, structures ranging from individual cells to those composed of few cells were dominant (see the first row of figure 1*a* and the range of area in the first row of figure 2*a*,*b*, considering that each cell has approx. 8 µm^2^). At medium cellular density, individual cells, pairs and strings prevailed; relatively larger strings and streams emerged through time (see the second row of figures 1*a* and 2*a*,*b*). At high cellular density, the morphology of structures and size distribution varies drastically through time (from right-skewed at 0 h to left-skewed at 8 h; the third row of figure 2*a*,*b*). It starts from droplets, streams and layered mounds at 0 h, then these merge and dissolve into and from branched streams and droplets at 4 h, and at 8 h, end up forming a single network that occupies an area five orders of magnitude larger than the small structures in the sample (the third row of figures 1*a* and 2*a*,*b*; electronic supplementary material, videos S7–S9).

### (c) Different types of cellular structures promote different types of motility at the structure and population levels

**Figure 3.**
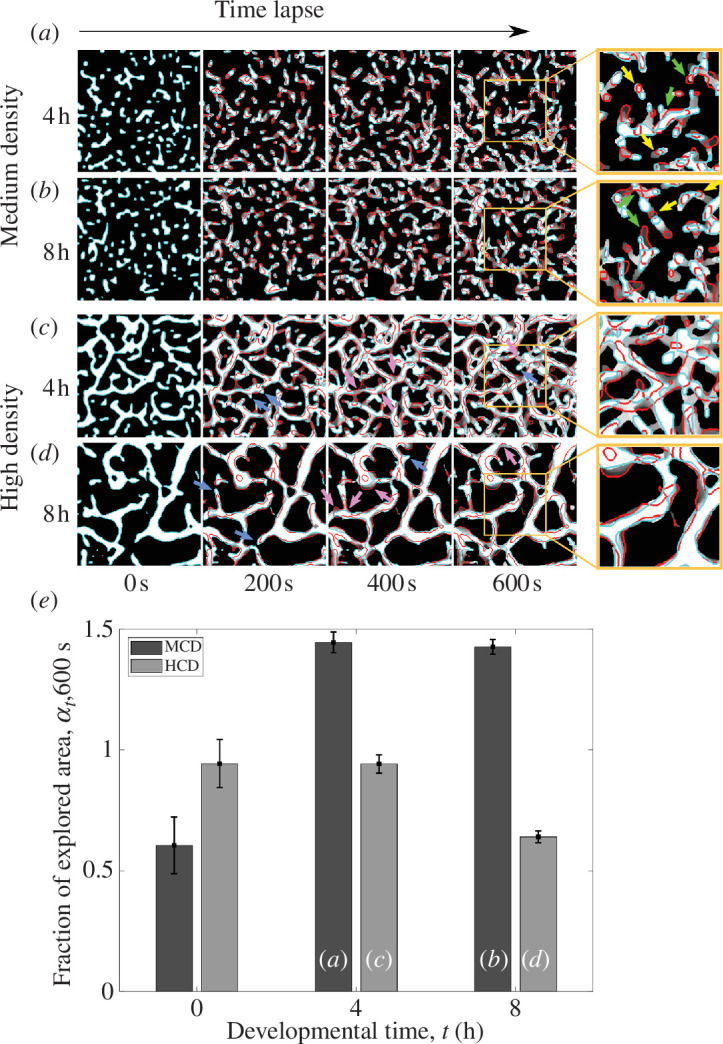
Dynamics of cellular structures at the population scale. (*a–d*) Medium and high cellular densities through the time-lapse, at developmental times *t* = 4 and 8 h. The images in the first column show the cell structures in white delimited with cyan at the time-lapse 0 s. These initial structures vary in shape, size and position through time, as illustrated in the second, third and fourth columns corresponding to time-lapses 200, 400 and 600 s. The structures at each given time are shown in white delimited by cyan, and the initial cellular arrangement at 0 h is delimited by red. Greyscale traces illustrate the gradual shape transition from 0 s to the time-lapse, going from darker values at earlier stages to white at the final time point. Figures at the rightmost column correspond to an enlarged small section of the figure at 600 s. Yellow and green arrows in (*a,b*) indicate the motility pattern of small and medium size groups, and blue and magenta arrows in (*c,d*) indicate growing and stretching zones. (*e*) Fraction of explored area (see the definition of $a_{t,T}$ in the main text) along *T* = 600 s for the medium and high cell densities at *t* = 0, 4 and 8 h. Letters in the columns indicate the corresponding conditions depicted in the figures above.

In order to explore how the prevalence of different structures impacts cellular dynamics across levels of organization, we further describe the structures’ dynamics and quantify the area explored by cells and structures at the whole population level. Small to medium-sized structures can move as a whole over empty space (e.g. the groups indicated by yellow arrows in figure 3*a*,*b*) or elongate in different directions, stretching and breaking into parts (green arrows). In contrast, large networks tend to confine the dynamics of cells, resulting in more stable structures, even though their shape is constantly changing due to spontaneously growing zones (indicated by blue arrows in figure 3*c*,*d*) or stretching zones (indicated by magenta arrows). As expected from the description above, high cell density populations exhibit lower values of the fraction of explored area $\left(a_{t,T}\right)$ than those at medium cell density. For medium cell density populations, $a_{t,T}$ shows a similar value for 4 and 8 h, while for the high cell density populations, there is an abrupt decay at 8 h. This aligns with the morphological changes observed at these two times, suggesting that large networks constrain cellular exploration. Overall, the behaviour of the cellular structures modifies the way in which cells explore the space and, thus, the population patterns.

## 4. Discussion and conclusions

We describe the cellular structures that arise dynamically during the early stages of aggregation in *M. xanthus* development at low cellular densities (figure 1). These structures exhibit an ample range of sizes, morphologies, behaviours and boundary stability. By quantifying the prevalence of these structures at different times, we uncover sequences and transitions in the prevalence of specific structures (figures 2 and 3). We also show how the sequences and transitions of different structures depend on cellular density. Notably, changes in the prevalence of cellular structures are associated with changes in patterns and motility at the population scale (figure 3).

We propose a typology for the different cellular structures we observe (figure 1). It is important to acknowledge that these structures are not static or discrete entities but rather dynamic, constantly changing in size and shape, often merging with or dissociating from other structures. Although ephemeral structures tend to be substituted by larger or more permanent ones, the transitions between them are density-dependent and do not strictly follow a determined sequence (figures 2 and 3). Moreover, small and large structures can coexist for the whole time-lapse (figure 3*b*). Without considering intermediate structures or spatiotemporal dynamics, development can be simplistically understood as a linear sequence of increasing size and stability. Here, we deepen into the richness of cellular aggregative morphologies, behaviour and the dynamics that unfold from development. In contrast with a view in which these structures sequentially follow each other every time, we show that they can change back and forth and can follow diverse trajectories while overall forming larger and steadier structures.

Studying *M. xanthus* behaviour and aggregation at low densities allowed us to characterize understudied types of structures such as ‘networks’, ‘strings’ or ‘droplets’, which might be relevant to bacterial dispersion or aggregation in diverse ecological contexts. We provide evidence to distinguish between these structures in general based on their size, shape and behaviour (e.g. electronic supplementary material, video S10). However, the proposed typology can be further tested and improved through the quantification of the structures’ dynamics, which can help clarify the distinction between some structures, such as large strings and thin streams.

Our results align with previous evidence showing that even at higher densities, not all cells form steady aggregates, but some disperse instead [17,20]. They are also in agreement with previous studies showing and studying similar aggregation patterns in *M. xanthus* [15,21]. However, we also find interesting differences between these studies. Thutupalli *et al*. [15] work with a WT and a non-reversing mutant strain that show significant differences in the formation of streams, the former having larger streams with steadier shapes than the latter. The aggregation dynamics and structures of the WT strain we use resemble more their mutant strain, although its ability to reverse is not impaired. In the case of Starruß *et al*. [21], they observe patterns of aggregation and stream formation that are surprisingly similar to those we see, but they study *M. xanthus* dynamics in a substrate with nutrients, that is, not in its developmental regime. From this, we can conclude that the structures and aggregative dynamics in *M. xanthus* cannot be directly associated with a particular genotype or environment, but that they depend on the interaction of the specific strain with its medium. On the same line, our findings emphasize that starvation or single-cell motility features are not sufficient to trigger *M. xanthus* aggregation but that cell density and probably other physical and cellular environmental conditions have to be met or produced by cells themselves.

Moreover, our experimental design allowed us to test the effect of initial densities on structure formation and aggregation dynamics. Changes in cellular density lead to qualitative changes in aggregation trajectories and the prevalence of the different types of cellular structures. This holds ecological significance because the density of *M. xanthus* in natural environments can be much lower than densities usually used in laboratory conditions and can vary with substrate textures, topography, liquid content, nutrient availability, among other biological and physicochemical factors [22–25]. Cellular density within a population is often heterogeneous, as both physical and cellular environmental conditions fluctuate through time and space as cells move and organize into different structures. Indeed, local density appears to be central in determining the behaviour of cells and groups of cells [17], but its effect on groups of different structures remains to be further explored.

Our findings regarding the role of cellular density can help us further understand the ecological conditions, including population density, in which aggregative multicellularity may have emerged in the evolutionary history of life. They can also help us understand how it may continue to arise when challenged by external stimuli or in interaction with different environments, including clinically relevant ones. Our results can indeed inform and create dialogue with clinical studies aiming to manage infection control and treatment of bacterial populations by understanding the coexistence and development of spatial and temporal structures during biofilm formation [26–28].

In conclusion, our experiments and analyses allowed us to uncover and begin to understand a wide range of cellular structures that arise during the aggregative development of *M. xanthus*, further unravelling specific aspects of this route to development and contributing to a nuanced understanding of the branched and diverse trajectories in which aggregation can occur in different cellular and ecological conditions, not only in *M. xanthus* but also in other aggregative multicellular groups.

## Data Availability

Data are available from the Dryad Digital Repository [29]. Supplementary material is available online [30].

## References

[1] Ginot F, Theurkauff I, Detcheverry F, Ybert C, Cottin-Bizonne C. 2018 Aggregation–fragmentation and individual dynamics of active clusters. Nat. Commun. **9**, 696. (10.1038/s41467-017-02625-7) 29449564 PMC5814572

[2] Arias Del Angel JA, Nanjundiah V, Benítez M, Newman SA. 2020 Interplay of mesoscale physics and agent-like behaviors in the parallel evolution of aggregative multicellularity. EvoDevo **11**, 1–18. (10.1186/s13227-020-00165-8) 33062243 PMC7549232

[3] Fu Y, Yu H, Zhang X, Malgaretti P, Kishore V, Wang W. 2022 Microscopic swarms: from active matter physics to biomedical and environmental applications. Micromachines **13**, 295. (10.3390/mi13020295) 35208419 PMC8876490

[4] Ben Amar M, Ciarletta P, Haas PA. 2023 Morphogenesis in space offers challenges and opportunities for soft matter and biophysics. Commun. Phys. **6**, 150. (10.1038/s42005-023-01242-9)

[5] Newman SA, Benítez M, Bhat R, Glimm T, Kumar KV, Nanjundiah V, Nicholson DJ, Sarkar S. 2024 Agency in the evolutionary transition to multicellularity. EcoEvoRxiv. (10.32942/X2X895)

[6] Vernerey FJ *et al*. 2019 Biological active matter aggregates: inspiration for smart colloidal materials. Adv. Colloid Interface Sci. **263**, 38–51. (10.1016/j.cis.2018.11.006) 30504078

[7] Niklas KJ, Newman SA. 2016 Multicellularity: origins and evolution. Cambridge, MA, USA: The MIT Press eBooks.

[8] Herron MD, Conlin PL, Ratcliff WC. 2022 The evolution of multicellularity. Boca Raton, FL, USA: CRC Press.

[9] Whitworth DE. 2008 Myxobacteria: multicellularity and differentiation. Washington, DC, USA: ASM press. (10.1128/9781555815677)

[10] Zhang Y, Ducret A, Shaevitz J, Mignot T. 2012 From individual cell motility to collective behaviors: insights from a prokaryote, Myxococcus xanthus. FEMS Microbiol. Rev. **36**, 149–164. (10.1111/j.1574-6976.2011.00307.x) 22091711

[11] Muñoz-Dorado J, Marcos-Torres FJ, García-Bravo E, Moraleda-Muñoz A, Pérez J. 2016 Myxobacteria: moving, killing, feeding, and surviving together. Front. Microbiol. **7**, 781. (10.3389/fmicb.2016.00781) 27303375 PMC4880591

[12] Arias Del Angel JA, Escalante AE, Martínez-Castilla LP, Benítez M. 2017 An evo-devo perspective on multicellular development of myxobacteria. J. Exp. Zool. B Mol. Dev. Evol. **328**, 165–178. (10.1002/jez.b.22727) 28217903

[13] Zhang H, Vaksman Z, Litwin DB, Shi P, Kaplan HB, Igoshin OA. 2012 The mechanistic basis of Myxococcus xanthus rippling behavior and its physiological role during predation. PLoS Comput. Biol. **8**, e1002715. (10.1371/journal.pcbi.1002715) 23028301 PMC3459850

[14] Peruani F, Starruß J, Jakovljevic V, Søgaard-Andersen L, Deutsch A, Bär M. 2012 Collective motion and nonequilibrium cluster formation in colonies of gliding bacteria. Phys. Rev. Lett. **108**, 098102. (10.1103/PhysRevLett.108.098102) 22463670

[15] Thutupalli S, Sun M, Bunyak F, Palaniappan K, Shaevitz JW. 2015 Directional reversals enable Myxococcus xanthus cells to produce collective one-dimensional streams during fruiting-body formation. J. R. Soc. Interface **12**, 20150049. (10.1098/rsif.2015.0049) 26246416 PMC4535398

[16] Liu G, Patch A, Bahar F, Yllanes D, Welch RD, Marchetti MC, Thutupalli S, Shaevitz JW. 2019 Self-driven phase transitions drive Myxococcus xanthus fruiting body formation. Phys. Rev. Lett. **122**, 248102. (10.1103/PhysRevLett.122.248102) 31322369

[17] Murphy P, Comstock J, Khan T, Zhang J, Welch R, Igoshin OA. 2023 Cell behaviors underlying Myxococcus xanthus aggregate dispersal. Msystems **8**, e0042523. (10.1128/msystems.00425-23) 37747885 PMC10654071

[18] Yang Z, Higgs P. 2014 Myxobacteria: genomics, cellular and molecular biology. Norfolk, UK: Caister Academic Press.

[19] Kaiser D, Crosby C. 1983 Cell movement and its coordination in swarms of Myxococcus xanthus. Cell Motil. **3**, 227–245. (10.1002/cm.970030304)

[20] Rossine FW, Martinez-Garcia R, Sgro AE, Gregor T, Tarnita CE. 2020 Eco-evolutionary significance of ‘loners.’ PLoS Biol. **18**, e3000642. (10.1371/journal.pbio.3000642) 32191693 PMC7081983

[21] Starruß J, Peruani F, Jakovljevic V, Søgaard-Andersen L, Deutsch A, Bär M. 2012 Pattern-formation mechanisms in motility mutants of Myxococcus xanthus. Interface Focus **2**, 774–785. (10.1098/rsfs.2012.0034) 24312730 PMC3499129

[22] Velicer GJ, Kroos L, Lenski RE. 1998 Loss of social behaviors by Myxococcus xanthus during evolution in an unstructured habitat. Proc. Natl Acad. Sci. **95**, 12376–12380. (doi:0027-8424/98/9512376-5$2.00/0) 9770494 10.1073/pnas.95.21.12376 PMC22839

[23] Kadam SV, Velicer GJ. 2006 Variable patterns of density-dependent survival in social bacteria. Behav. Ecol. **17**, 833–838. (10.1093/beheco/arl018)

[24] Rivera-Yoshida N, Hernández-Terán A, Escalante AE, Benítez M. 2020 Laboratory biases hinder eco-evo-devo integration: hints from the microbial world. J. Exp. Zool. B Mol. Dev. Evol. **334**, 14–24. (10.1002/jez.b.22917) 31829529

[25] Ramos CH, Rodríguez-Sánchez E, Del Angel JAA, Arzola AV, Benítez M, Escalante AE, Franci A, Volpe G, Rivera-Yoshida N. 2021 The environment topography alters the way to multicellularity in Myxococcus xanthus. Sci. Adv. **7**, eabh2278. (10.1126/sciadv.abh2278) 34433567 PMC8386931

[26] Bidossi A, Bottagisio M, Savadori P, De Vecchi E. 2020 Identification and characterization of planktonic biofilm-like aggregates in infected synovial fluids from joint infections. Front. Microbiol. **11**, 1368. (10.3389/fmicb.2020.01368) 32714301 PMC7344156

[27] Knott S *et al*. 2021 Staphylococcus aureus floating biofilm formation and phenotype in synovial fluid depends on albumin, fibrinogen, and hyaluronic acid. Front. Microbiol. **12**, 655873. (10.3389/fmicb.2021.655873) 33995317 PMC8117011

[28] Rivera-Yoshida N, Bottagisio M, Attanasi D, Savadori P, De Vecchi E, Bidossi A, Franci A. 2022 Host environment shapes S. aureus social behavior as revealed by microscopy pattern formation and dynamic aggregation analysis. Microorganisms **10**, 526. (10.3390/microorganisms10030526) 35336102 PMC8949161

[29] Rivera-Yoshida N, Arzola AV, Beníıtez M. 2024 Data from: Unravelling a diversity of cellular structures and aggregation dynamics during the early development of Myxococcus xanthus. Dryad Digital Repository. (10.5061/dryad.zcrjdfnnk) PMC11496945 39439355

[30] Rivera-Yoshida N, V. Arzola A, Benítez M. 2024 Data from: Unravelling a diversity of cellular structures and aggregation dynamics during the early development of Myxococcus xanthus. Figshare. (10.6084/m9.figshare.c.7477948) PMC11496945 39439355

